# Multimodal Imaging in Susac Syndrome: A Case Report and Literature Review

**DOI:** 10.3390/ijerph18073435

**Published:** 2021-03-26

**Authors:** Simone Alex Bagaglia, Franco Passani, Giovanni William Oliverio, Leandro Inferrera, Feliciana Menna, Alessandro Meduri, Cosimo Mazzotta

**Affiliations:** 1 Department of Ophthalmology, University of Siena, Policlinico Santa Maria alle Scotte, Viale Mario Bracci, 53100 Siena, Italy; dott.bagaglia@gmail.com; 2 Department of Surgery Section of Ophthalmology USL Toscana Nord Ovest, Civic Hospital of Carrara, 54033 Carrara, Italy; f.passani@usl1.toscana.it; 3 Biomedical, Dental and Morphological and Functional Images Sciences, Department of Ophthalmology, University of Messina, 98124 Messina, Italy; inferreraleandro@gmail.com (L.I.); ameduri@unime.it (A.M.); 4 Head and Neck Department, University of Naples “Federico II”, 80131 Naples, Italy; feliciana.menna@gmail.com; 5 Siena Crosslinking Center, 53100 Siena, Italy; cgmazzotta@libero.it

**Keywords:** susac syndrome, multimodal imaging, optical coherence tomography angiography, retinal branch artery occlusion, fluorescein angiography

## Abstract

Susac syndrome (SS) is a rare microangiopathy that involves arterioles of the brain, retina, and cochlea. Diagnosis is extremely difficult because of the rarity of the disease and because the signs and symptoms often occur at different times. Multidisciplinary approaches and multimodal images are mandatory for diagnosis and prompt therapy. In this report, we describe a case of SS and the application of multimodal retinal imaging to evaluate the ophthalmologic changes and to confirm diagnosis. Early diagnosis and therapy based on the associations of steroids and immunosuppressants are necessary to limit the sequelae of the disease.

## 1. Introduction

Susac syndrome (SS) is a rare autoimmune syndrome characterized by microvascular alterations involving the precapillary arterioles of the brain, retina, cochlea, and semicircular canals [1]. This disease affects mainly women, and the age of onset ranges from 9 to 58 years [2,3]. Laboratory investigations, brain magnetic resonance imaging (MRI), fluorescein angiography (FA), and audiometry findings enable its diagnosis [4]. Diagnosis is extremely difficult as the disease is particularly rare and because the signs and symptoms often occur at different times. Multidisciplinary approaches and multimodal imaging are frequently used to confirm SS diagnosis. Brain magnetic resonance imaging (BMRI) findings are not specific and could be suggestive of several neurological diseases. Moreover, prompt treatment including corticosteroids and immunosuppressants is required to limit complications of the disease [5]. Retinal branch artery occlusion (BRAO), vascular leakage, and arteriolar wall hyper-fluorescence (glass plaques) are the primary ophthalmic findings and are conventionally seen in FA and wide-field color fundus photography. Optical coherence tomography (OCT) improved the evaluation of the retinal layers and may also help differentiate SS from other retinal diseases [6,7,8]. Optical coherence tomography angiography (OCTA) is a novel noninvasive imaging technique that provides retinal and choroidal volumetric bold flow data, permitting retinal vessel reconstruction for imaging of retinal perfusion and function [9,10,11,12].

In this paper, we considered the application of the multimodal retinal imaging approach to describe a case of SS.

## 2. Case Report

**Figure 1:**
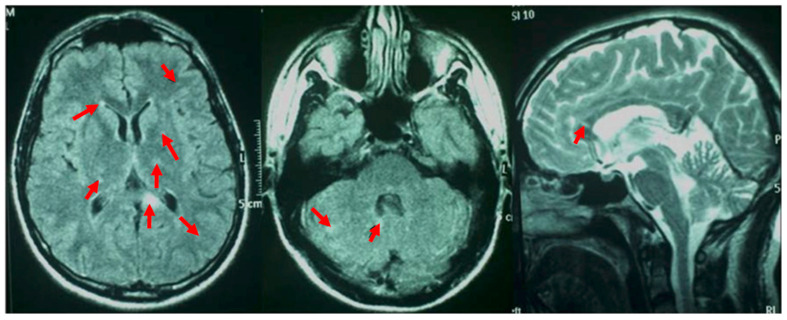
Magnetic resonance of the brain: the red arrows show various rotund and ellipsoid focal lesions involving the corpus callosum, periventricular and subcortical white matter, deep grey matter (basal ganglia and thalami), and cerebellum.

**Figure 2:**
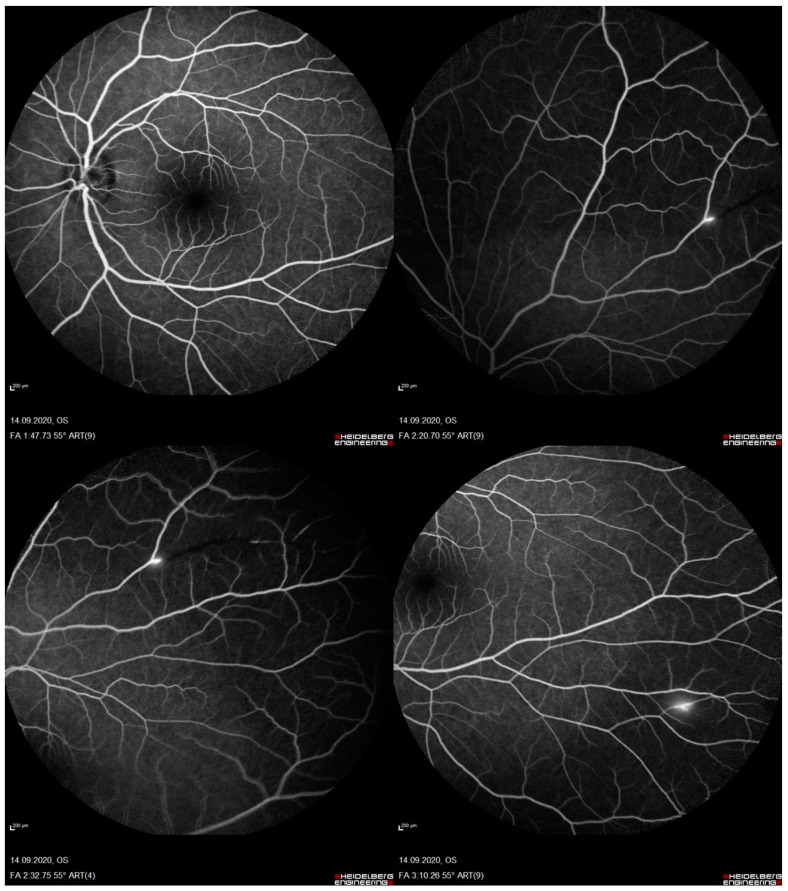
Fluorescein angiography examination exhibiting branch retinal artery occlusion in the left eye.

**Figure 3:**
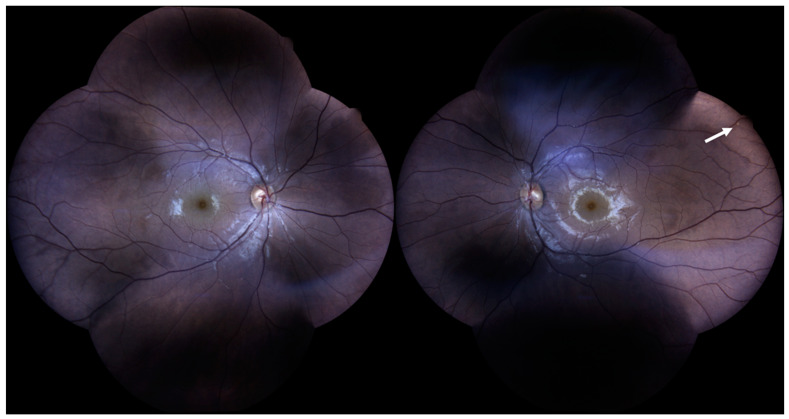
Color fundus photography revealing a glass plaque (white arrow) as yellowish lipid sediments at the mid-segment of the retinal arterioles in the left eye.

**Figure 4:**
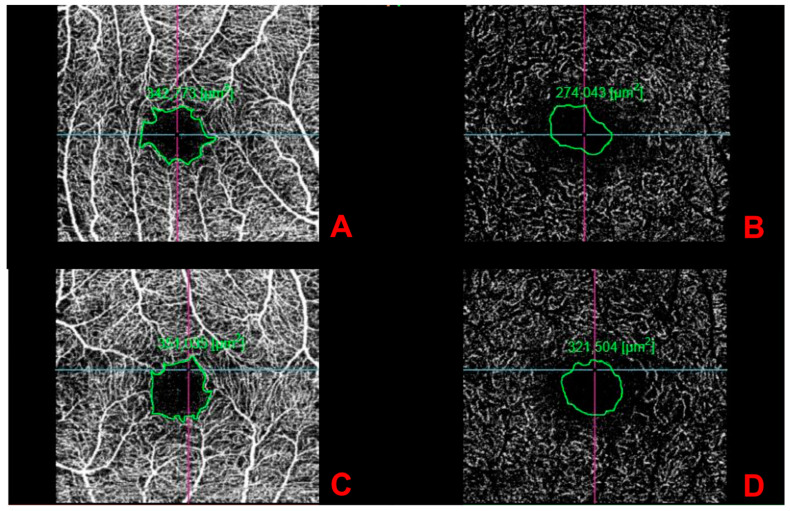
Foveal avascular zone enlargement in optical coherence tomography angiography images of the superficial and deep layers (**A**,**B**) right eye; (**C**,**D**) left eye).

A 33-year-old man was admitted to the emergency unit for headaches, a referred visual field loss, dizziness, and weakness of the limbs. The patient had a history of malignant external otitis [13], hearing loss, and cochlear implant surgery [14,15,16] and used a mandibular advanced device for obstructive sleep apnea syndrome [17]. Neurological evaluation demonstrated a decrease in attention and memory, ataxia, dysmetria, and mild weakness of the extremities. Although the brain CT scans were normal, MRI revealed numerous rounds and focal lesions in the corpus callosum, subcortical white matter, deep grey matter (basal ganglia and thalami), and cerebellum (Figure 1). Additionally, laboratory analyses were normal while anti-smooth muscle antibodies (ASMA), anti-nuclear antibodies (ANA), and anti-cardiolipin IgM antibodies were positive. Two years ago, the patient underwent photorefractive refractive keratectomy (PRK) for myopia [18]. His best-corrected visual acuity (BCVA) was 20/20 Snellen in both eyes, with spherical equivalent refractions of −0.75 in the right eye and of −0.50 in the left eye. The intraocular pressure was 16 mmHg in both eyes (Goldmann Applanation Tonometer). Anterior segment examination was normal in both eyes; however, fundus evaluation showed ischemic retinal whitening in the inferior-temporal and nasal periphery outside the vascular arcades, related to BRAO. FA was performed by a Heidelberg retina angiograph (HRA II, Heidelberg Engineering, Dossenheim, Germany), showing in both eyes arterial occlusion with vasculitis signs (Figure 2). Finally, considering the clinical, laboratory, serological, and neuro-radiological findings, SS was diagnosed. Further ophthalmologic investigations were conducted; in particular, the patient underwent multimodal retinal imaging including wide-field color fundus photography acquired using a non-mydriatic fundus camera (Canon CR-2, Tokyo, Japan) and high-definition optical coherence tomography angiography (OCTA, Angio OCT Scans, Heidelberg Engineering, Heidelberg, Germany). Color fundus photography confirmed a slight area of retinal ischemia observed in a fundus examination conducted first and revealed the existence of glass plaques as yellowish lipid sediments at the mid-segment of the retinal arterioles (Figure 3). The autofluorescence images did not show specific alterations in physiological retinal autofluorescence. The OCTA scans revealed decreased vascular perfusion in correspondence with the ischemic area, previously observed in FA, as well as an increased foveal avascular zone (FAZ) area in both superficial and deep vascular plexuses (Figure 4). OCT also showed a reduction in the formation of the inner limiting membrane to internal nuclear layer areas.

## 3. Discussion

Although the clinical trio including focal neurological deficits, hearing defeat, and retinal arterial occlusions is considered pathognomonic, the whole clinical manifestation occurs in only 13% of patients at onset [1,2,3]. The most suggestive neurological symptoms and clinical signs suggestive of brain involvement are changes in consciousness or new cognitive deficiency or behavioral modifications, new focal neurological symptoms, and headaches [18,19]. Susac et al. defined a neuroimaging triad of white matter lesions in the corpus callosum, deep grey matter alterations, and leptomeningeal enhancement. Distinctive neuroimaging alterations, defined as T2/fluid-attenuated inversion recovery hyper-intense multifocal, round brain lesions with at least one centrally set in the corpus callosum, are definitive [18,19]. Additionally, their dimensions and forms are inconstant, including square, triangular, or rectilinear lesions. Certain brain involvement is described as the occurrence of at least one of the clinical manifestations in addition to the characteristic MRI findings [18,19]. These lesions are secondary to arteriolar infarction in the callosum and, afterwards, cavitate and change into manifestation of a hole. Symptoms correlated with retinal ischemia could manifest as a visual field altitudinal defect or central-paracentral scotoma [18]. However, in the case of a far periphery of retina involvement, patients could be asymptomatic. In some patients, the encephalopathy could be severe and visual symptoms may not be referred even though ophthalmologic examination is mandatory if Susac syndrome is suspected, even in asymptomatic patients [19]. The fundus examination may reveal the presence of Gass plaques as yellow reflective lesions due to an autoimmune local reaction in the retinal arterial wall and characteristic retinal whitening in the area of BRAO [20,21,22,23]. However, Gass plaques are characteristic but not pathognomonic of Susac syndrome and could be observed in various retinal disorders such as Eale’s disease and retinal lymphoma. Furthermore, vascular alteration is common in the early phases of the disease and could then disappear [19]. FA is able to document the vascular wall damage as arteriolar hyper-fluorescence at the site of infarction [4]. MRI and FA remain the bases of diagnostic assessment [18,19]. Spectral-domain optical coherence tomography (SD-OCT) is able to evaluate the microstructure alterations of each individual retinal layer, documenting the ischemic swelling and better analyzing the retinal changes compared to FA, but it does not investigate vascular perfusion [6]. OCTA is a safe, recent, dye-less, and fast instrumental examination able to acquire high-resolution images of retinal microcirculation [10]. In this case, we report the microvascular findings in a patient affected by acute BRAO and correlating the OCTA features with those of wide-field color fundus photography and FA. We highlighted alterations in both superficial and deep retinal vascular plexuses, such as a low flow area, in the course of BRAO and reported a reduction in macula thickness in OCT images in the formation of the inner limiting membrane to internal nuclear layer areas. Some authors reported specific alterations such as a reduction in retinal autofluorescence in the ischemic area [11]; in our case, we did not observe any specific alteration in retinal autofluorescence. Furthermore, SS is an important differential diagnosis in numerous cerebrovascular conditions, and early identification supports starting prompt treatment, reducing relapses, and improving outcomes [5]. Although FA is necessary for identification of the disease, this is a time-consuming, dye-dependent technique and does not allow users to examinate the deep capillary plexus. OCTA may be an effective alternative to the standard FA as it consents to the microvascular analysis of both superficial and deep capillary plexi and to monitoring of the vascular density changes without dye injection [24]. However, additional prospective studies are necessary to establish the role of OCTA, wide-field color fundus photography, and AF to monitor disease activity and to determine the efficacy of new therapeutic approaches [25].

## 4. Conclusions

In conclusion, MRI and FA are the main diagnostic instruments and may lead clinical ophthalmologists to the correct diagnosis of SS; however, new diagnostic instruments such as OCTA and wide-field color retinal photography may provide an advantage in early diagnosis and follow-up of SS, offering an effective alternative to standard FA; in evaluating microvascular changes in all capillary plexuses; and in monitoring retinal vessel density alterations during prompt therapy without dye injection.

## Data Availability

The data used to support the findings of this study are available from the corresponding author upon request.
